# A Comparison dataset on static test using two concentrations of hydrogen peroxide for prediction of acid mine drainage

**DOI:** 10.1016/j.dib.2023.109706

**Published:** 2023-10-20

**Authors:** Muhammad Sonny Abfertiawan, Yoseph Palinggi, Mindriany Syafila, Marisa Handajani, Kris Pranoto

**Affiliations:** a Water and Wastewater Engineering Research Group, Faculty of Civil and Environmental Engineering, Bandung Institute of Technology, Indonesia; b Environmental Department, Kaltim Prima Coal, Indonesia

**Keywords:** Sulfide mineral, Static test, Net acid generating, Acid-base accounting, Acid mine drainage

## Abstract

Acid mine drainage (AMD) is a serious problem in many coal and mineral mines. Over the past 50 years, many researchers have developed static tests that play an essential role in preventing AMD. In Indonesia, static tests are conducted using two methods: acid–base accounting (ABA) and net acid generation (NAG) tests. On an operational scale, mining companies commonly use the NAG test because it is simpler and faster than ABA. The NAG test is performed using hydrogen peroxide at a concentration of 15% as a strong oxidizing agent, according to the ARD Test Handbook and Indonesian National Standard (SNI). However, since 1990, an Indonesian coal mining company, PT Kaltim Prima Coal, is conducting NAG tests using 7.5% hydrogen peroxide. In the present dataset, we compared the results of NAG tests obtained using 7.5% and 15% hydrogen peroxide for 564 rock core samples. The dataset also shows the behavior of the NAG solution for each representative rock characteristic—including the concentrations of Fe, Mn, and SO_4_, oxidation reduction potential (ORP), conductivity, total dissolved solids (TDS), and temperature—during the test. This dataset can be useful for researchers to investigate the differences in the NAG test when 7.5% and 15% hydrogen peroxide are used, as well as to understand the oxidation behavior of sulfide minerals when hydrogen peroxide is used as a strong oxidizing agent for AMD.

Specifications Table

**Table:** 

Subject	Environmental Chemistry
Specific subject area	Mine water geochemistry/acid mine drainage
Data format	Raw
Type of data	Table
Data collection	Rock core samples (*n* = 564) representing non-acid and potentially-acid forming rocks from different lithologies were obtained by exploration activities at Bengalon and Sangatta mine sites. Static tests were carried out according to the ARD Test Handbook, Indonesian National Standard (SNI), and PT Kaltim Prima Coal standards. The samples were prepared in advance: pulverized using a jaw crusher and subjected to splitter and oven equipment to obtain a representative sample (2.5 g) of particle size 200 mesh or <75 µm. After adding H_2_O_2_, the NAG solution was analyzed for metal (Fe, Mn, and Al) and SO_4_^2−^ contents and temperature changes. Spectrophotometer and thermometer were used according to the Standard Methods of Analysis of Water and Waste from the American Public Health Association (APHA).
Data source location	Site Location: Bengalon and Sangatta Mine Sites Coordinate: 117°27’7.40”–117°40’43.40” E and 0°31’20.52”–0°52’4.60” N Company: PT Kaltim Prima Coal Region and Province: East Kutai Region, East Kalimantan Province Country: Indonesia
Data accessibility	Repository name: Mendeley Data Data identification number: doi: 10.17632/kdrk22t63z.1 Direct URL to data: https://data.mendeley.com/datasets/kdrk22t63z/1
Related research article	M.S. Abfertiawan, Y. Palinggi, M. Syafila, M. Handajani, K. Pranoto, The comparison of 7.5 and 15% hydrogen peroxide as oxidizing agent in static tests of acid mine drainage potential in Indonesia, Heliyon 9 (2023), e18687. https://doi.org/10.1016/j.heliyon.2023.e18687.

## 1 Value of the Data

• This dataset can help researchers understand the differences in static tests on rock samples using 7.5% and 15% hydrogen peroxide as strong oxidizing agents [1].
• This dataset also shows the characteristics of the metal concentration and temperature in the NAG solution when different concentrations of hydrogen peroxide are added.
• This dataset can be helpful to researchers conducting studies on the oxidation of sulfide minerals in AMD generation.
• This dataset can be reused to study the critical factors that determine AMD generation and its oxidation products.

## 2 Data Description

The dataset is provided as an Excel file (2023 - Static Test - Mendeley Data.xlsx) [2] and includes three worksheets:

1. Sheet “Static Test Data”: This sheet includes columns for sample number, sample ID, NAG, and ABA tests. The ‘NAG test’ column is sub-divided into NAG results column for 7.5% and 15% hydrogen peroxide, each with a pH column—NAG at pH 4.5 and NAG at pH 7.0. The ‘ABA test’ column has data on ANC (Acid Neutralizing Capacity), TS (Total Sulfur), MPA (Maximum Potential Acid), and NAPP (Net Acid Producing Potential) test results.
2. Sheet “Solution Characteristic”: This sheet includes columns for ere are sample ID, pH, ORP (mV), conductivity (μg/L), TDS (mg/L), Fe (mg/L), and Mn (mg/L). Results are presented in columns for each test with 7.5% and 15% hydrogen peroxide.
3. Sheet “Solution Temperature”: This sheet includes columns for time (in min) which describe the reaction time, when 7.5% and 15% hydrogen peroxide is added, for up to 150 min of observation. Temperature changes were observed for four samples representing different rock characteristics.

## 3 Experimental Design, Materials, and Methods

### 3.1 Location

Rock samples were collected from Sangatta and Bengalon Mine Sites and taken to PT Kaltim Prima Coal, located in East Kutai Regency, East Kalimantan, Indonesia. The mining area is in the Miocene Balikpapan Formation (Early Middle Miocene – Early Late Miocene), known to have a thickness of 1,000 to 1,500 m, and was deposited in deltaic to coastal plain environments. The formation conformably overlies the Pulau Balang Formation and is locally interfingered with that formation. The dominant lithologies are sandstone, claystone, siltstone, shale, and coal.

### 3.2 Rock sampling and preparation

**Fig. 1:**
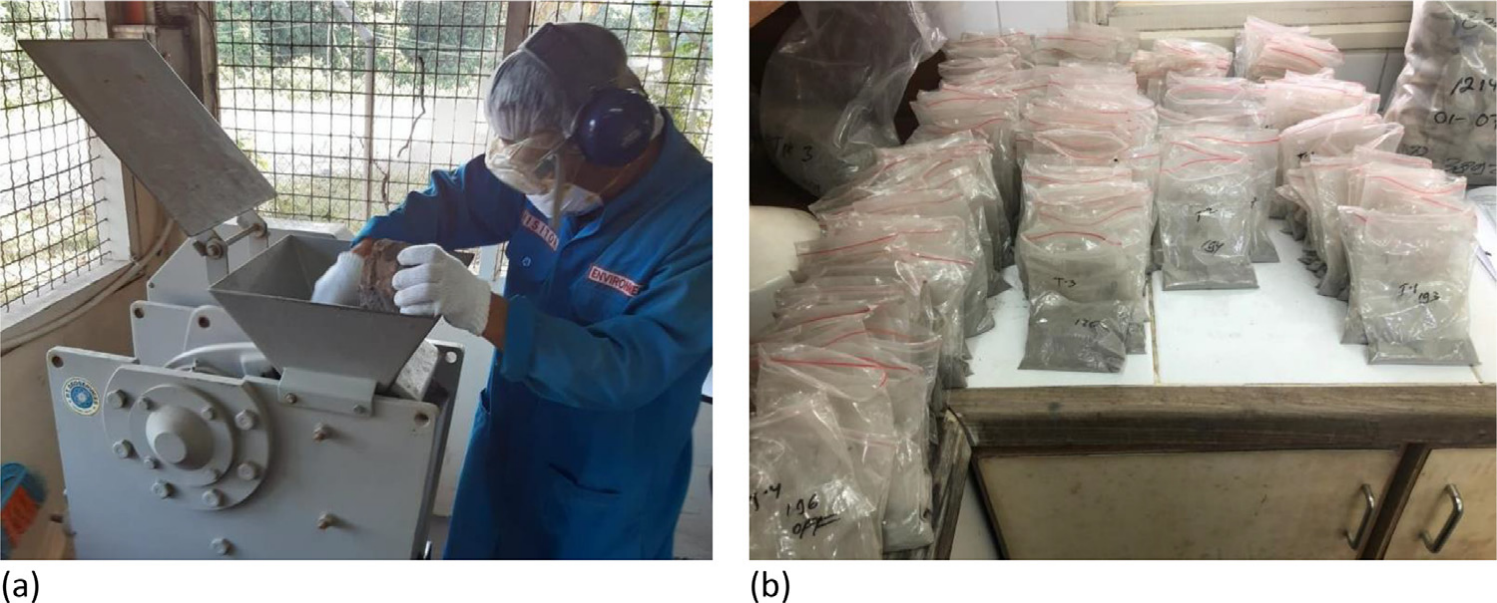
Sample preparation prior to AMD formation test. (a) Sample crushing activities using a jaw crusher. (b) Pulverized samples with a size of 200 mesh or <75 µm.

A total of 564 samples were obtained from rock cores (core diameter = six cm), with a density of 2.210–2.360 kg/m^3^, representing different lithologies. The samples were assessed to determine whether they belong to non-acid-forming (NAF) or potentially acid-forming (PAF) categories. The samples were crushed using a jaw crusher with an opening of 2 cm, to produce samples with smaller particle sizes. Furthermore, samples were taken representatively using a splitter and finely ground until the sample size reached 200 mesh or <75 µm. Samples (2.5 g) were taken according to the standard static test method for AMD generation (Fig. 1).

### 3.3 Static test methodology

The NAG test was performed using 7.5% and 15% H_2_O_2_ as the strong oxidizing agent of sulfide minerals, according to the standard operating procedures of the PT Kaltim Prima Coal, ARD Test Handbook, Amira International [3], and Indonesian National Standard (SNI) 6597:2011. Static tests were performed to determine the capacity for acid generation, including paste pH, TS, ABA, and NAG tests. The NAG test was performed according to the following procedure:

a. Pulverized rock core samples (2.5 g each), with a particle size of 200 mesh, were placed in 500 mL Erlenmeyer flasks.
b. To this, 250 mL of 7.5% or 15% hydrogen peroxide was added, separately. The flasks were placed in a fume hood overnight, for 12 h.
c. The solution was heated in an Erlenmeyer flask on a hotplate for approximately 30 min until boiling. Deionized water was added to maintain a constant initial volume.
d. The solution was allowed to cool down to room temperature (25 °C). The pH of the solution was measured and recorded as the NAG pH.
e. The sample solution was then titrated with NaOH until the pH changed from 4.5 and 7. The procedure was performed under the following conditions.
  • If NAG pH > 2, then solution was titrated with 0.10 M NaOH.
  • If NAG pH = 2, then solution was titrated with 0.50 M NaOH.

The net acid-generating capacity was calculated using the following equation:

$$NAG=\frac{4q\;x\;V\;x\;M}{W}$$

Where:

NAG = Net Acid Generating Capacity (kg H_2_SO_4_ /t)

V = volume of NaOH used in titration (mol/L)

M = concentration of NaOH used in titration (mol/L)

W = weight of sample reacted (g)

**Fig. 2:**
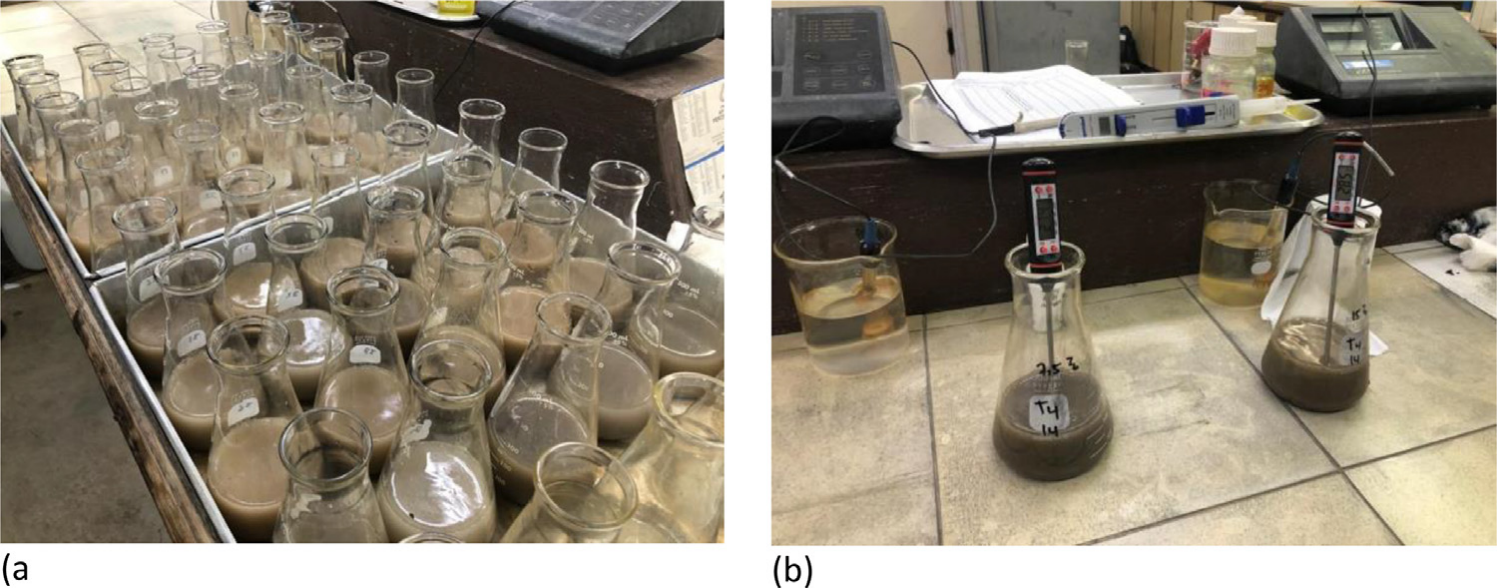
NAG Tests on 564 rock core samples conducted for determining acid mine drainage in PT Kaltim Prima Coal. (a) Samples after adding hydrogen peroxide (b) Solution temperature measurement.

Twenty rock samples representing different rock characteristics were subjected to an acid–base accounting (ABA) test. The ABA method involves two analyses: total sulfur and potential neutralization. TS was analyzed using the high-temperature combustion method to determine the maximum potential acidity (MPA) produced from the sample, using a LECO Carbon Sulfur Analyzer in the laboratory of PT Kaltim Prima Coal. The MPA calculation of the TS was performed using a stoichiometric approach, where 1% pyrite mineral was considered equivalent to 30.6 kg H_2_SO_4_/ton. The acid-neutralizing capacity (ANC) was determined by adopting the Sobek method (1978): a standard amount of hydrochloric acid was added to the sample and heated so that the reaction could take place quickly. The solution was titrated with standard sodium hydroxide to determine the amount of unreacted HCl. The amount of acid measured from this analysis was expressed in the same units as the MPA, i.e., kg H_2_SO_4_/ton. The difference between the MPA and ANC values was determined as the net acid-producing potential (NAPP). Tests were also conducted on the NAG solution after adding H_2_O_2_, by analyzing the concentrations of metals (Fe, Mn, and Al), SO_4_^2−^, and temperature changes. The test was conducted only on samples representing the rock characteristics (PAF and NAF). Pyrite is known to be one of the minerals which was detected in the PAF sample. All the mineralogical results can be found in ref. [1] (Fig. 2).

## Limitations

Acid–based accounting tests were not performed on all samples. The ABA test was performed only on five samples for each rock type.

## Ethics Statement

The authors have read and followed the ethical requirements for publication in *Data in Brief* and have confirmed that the current work does not involve human subjects, animal experiments, or any data collected from social media platforms.

## CRediT authorship contribution statement

**Muhammad Sonny Abfertiawan:** Conceptualization, Methodology, Validation, Visualization, Investigation, Resources, Data curation, Writing – original draft, Supervision, Project administration. **Yoseph Palinggi:** Conceptualization, Funding acquisition, Investigation, Data curation, Resources, Writing – review & editing, Supervision, Project administration. **Mindriany Syafila:** Conceptualization, Writing – review & editing, Supervision. **Marisa Handajani:** Conceptualization, Writing – review & editing, Supervision. **Kris Pranoto:** Conceptualization, Funding acquisition, Supervision.

## Data Availability

A Dataset on Static Test using 7.5% and 15% of Hydrogen Peroxide for Acid Mine Drainage Prediction (Original data) (Mendeley Data)
